# Multivariate analysis methods improve the selection of strawberry genotypes with low cold requirement

**DOI:** 10.1038/s41598-022-15688-4

**Published:** 2022-07-06

**Authors:** Eneide Barth, Juliano Tadeu Vilela de Resende, Keny Henrique Mariguele, Marcos Deon Vilela de Resende, André Luiz Biscaia Ribeiro da Silva, Sushan Ru

**Affiliations:** 1 Empresa de Pesquisa Agropecuária e Extensão Rural de Santa Catarina (Epagri), Rua XV de Novembro, 525, Pomerode, SC 89107-000 Brazil; 2 Departamento de Agronomia, Universidade Estadual de Londrina/UEL, Rodovia Celso Garcia, Km 380, Londrina, PR 86051-900 Brazil; 3 Empresa de Pesquisa Agropecuária e Extensão Rural de Santa Catarina (Epagri), Rodovia Antônio Heil, 6800, Itajaí, SC 88318-112 Brazil; 4 Departamento de Estatística, Embrapa Café/Universidade Federal de Viçosa, Campus Universitário, Viçosa, MG 36570-900 Brazil; 5 Department of Horticulture, Auburn University, 101 Funchess Hall, Auburn, AL 36849 USA

**Keywords:** Agricultural genetics, Plant genetics

## Abstract

Methods of multivariate analysis is a powerful approach to assist the initial stages of crops genetic improvement, particularly, because it allows many traits to be evaluated simultaneously. In this study, heat-tolerant genotypes have been selected by analyzing phenotypic diversity, direct and indirect relationships among traits were identified, and four selection indices compared. Diversity was estimated using K-means clustering with the number of clusters determined by the Elbow method, and the relationship among traits was quantified by path analysis. Parametric and non-parametric indices were applied to selected genotypes using the magnitude of genotypic variance, heritability, genotypic coefficient of variance, and assigned economic weight as selection criteria. The variability among materials led to the formation of two non-overlapping clusters containing 40 and 154 genotypes. Strong to moderate correlations were found between traits with direct effect of the number of commercial fruit on the mass of commercial fruit. The Smith and Hazel index showed the greatest total gains for all criteria; however, concerning the biochemical traits, the Mulamba and Mock index showed the highest magnitudes of predicted gains. Overall, the K-means clustering, correlation analysis, and path analysis complement the use of selection indices, allowing for selection of genotypes with better balance among the assessed traits.

## Introduction

World widely grown, strawberries (*Fragaria* × *ananassa* Duchesne) are popular fruits used for fresh or processing markets^1–3^. In 2020, strawberry production was 9 million tons, with China and United States contributing to more than 50% of this world production^4^. The most common strawberry cultivars in the world are often poorly adapted to tropical conditions as they are mainly developed by breeding programs in temperate regions, such as the United States, Spain, and Italy^5^. In South America, most strawberry seedlings are grown in nurseries of Argentina and Chile, significantly raising the cost of production in countries like Brazil, where strawberries are grown in 4,300 hectares and more than 75% of seedlings are imported from these nurseries^4^.

Strawberry cultivars are usually selected based on yield, resistance to pests and disease, adaptability to semi-hydroponic systems, and fruit quality such as firmness, sweetness, acidity, and aroma^6–8^. Assembling all the favorable characteristics into a single cultivar is complex, especially because strawberries have an octaploid genome highly interactive with the environment^9^. After making biparental crosses, strawberry cultivar development takes several rounds of selection to identify the best offspring as potential cultivars. The process of selecting among hundreds or thousands of seedlings is arduous, time-consuming, and costly. Traditionally, the selection is conducted for a single trait either through direct selection, which is directly based on the trait of interest, or indirect selection, which uses a correlated characteristic to select the trait of interest^8^. However, single trait selection may result in cultivars that do not adequately meet the demands of producers and consumers^10^. Combining direct and indirect effects can better evince the importance of traits on an independent variable, such as production of commercial fruits^11,12^. Using the path analysis to seek a better understanding of cause and effect, along with multivariate techniques, such as selection indices, to simultaneously evaluate multiple traits may be the best strategy for obtaining genotypes that balance agronomic, biometric, and biochemical characteristics, especially in the early stages of a breeding program^10,13,14^.

Thus, the variability of a population can be explored through cluster analysis, among other statistical tools. The K-means algorithm is a non-hierarchical data exploration method that maximizes the variation component between the formed K-groups while minimizing the variation within each group^15^. Still, determining the number of groups, which is defined a priori, is complex and can generate imprecise analyses. To minimize this possibility, the Elbow Method determines the ideal number of clusters^16^.

This study focused on the classic parametric index presented by Smith^17^ and Hazel^18^ and the base index established by Williams^19^. The classic Smith-Hazel index employs matrices of genetic and phenotypic variances and covariances estimated in the analysis of variance. This index consists of obtaining the maximum correlation between the genotypic aggregate (H) and the index (I). The H is a linear combination of the analyzed traits, pondered by a coefficient established by the economic weights previously assigned to each trait. The I consist of a linear combination of the “x” values of each trait, pondered by a coefficient to be estimated. The base index^19^ is established by the linear combination of the average phenotypic values of traits pondered directly by their respective economic weights. The index is estimated by: ${\text{I}}\, = \,{\text{a}}^{{1}} {\text{y}}^{{1}} \, + \,{\text{a}}^{{2}} {\text{y}}^{{2}} \, + \cdots + \,{\text{a}}^{{\text{n}}} {\text{y}}^{{\text{n}}} \, = \,{\text{y}}^{\prime}{\text{a}}$, where y_j_ is the mean of the jth trait and p_j_ the economic weight. As for the non-parametric indices, the sum of ranks from Mulamba and Mock^20^ and the genotype-ideotype^21^ indices were used. The index of Mulamba and Mock ranks genotypes in relation to each trait individually, assigning absolute values, according to the classification direction determined by the breeder, from highest to lowest, or vice versa, depending on how this direction favors the genetic improvement. The values assigned in the classification of each trait are added, resulting in the selection index. The genotype-ideotype index^21^ estimates the distance of the evaluated genotypes in relation to an ideotype previously defined by the breeder. The first step is to identify the favorable value for improvement based on the average, maximum, and minimum values informed by the statistical computer program. This favorable value, called optimal value (OVj), must be within an upper (UL) and lower (LL) limit for each trait (LL_j_ ≤ X_ij_ ≤ UL_j_). The OVj is corrected by a constant concerning the depreciation of the genotype average, C_j_ (C_j_ = UL_j_−L_j_), resulting in the Y_ij_ value. This process guarantees that any value of X_ij_ that is outside the optimal range is not selected. Subsequently, the Y_ij_ values obtained with the transformation are standardized and pondered by weights previously assigned by the breeder for each trait. The OVj for each trait is standardized and pondered.

Overall, studies have shown that applying the aforementioned selection indices, which associate information from various characters, increases the success rate in crop improvement programs, including alfalfa^22^, passion fruit^23^, soybean^24,25^, sweet potato^26^, among others. Nevertheless, the use of indices in the strawberry genetic improvement process has been just recently shown in the literature^13^ and still needs to be better understood. The objective of this study was to evaluate and select intraspecific strawberry genotypes, to assess their phenotypic diversity, to compare different selection indices, and determine the direct and indirect relationship among yield and biochemical traits, using multivariate analysis methods.

## Results

**Figure 1:**
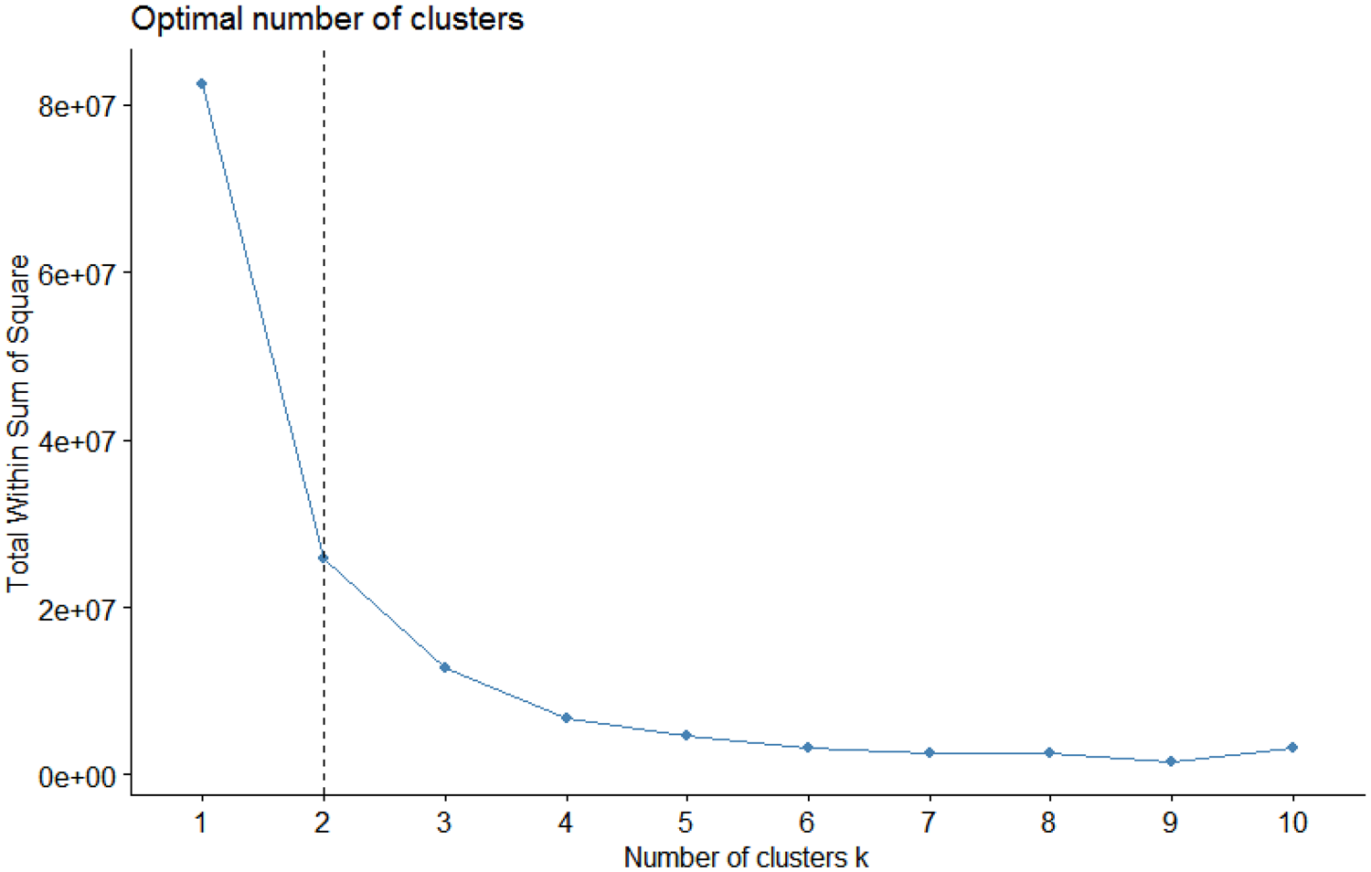
Identification of the elbow point for the evaluated dataset.

**Figure 2:**
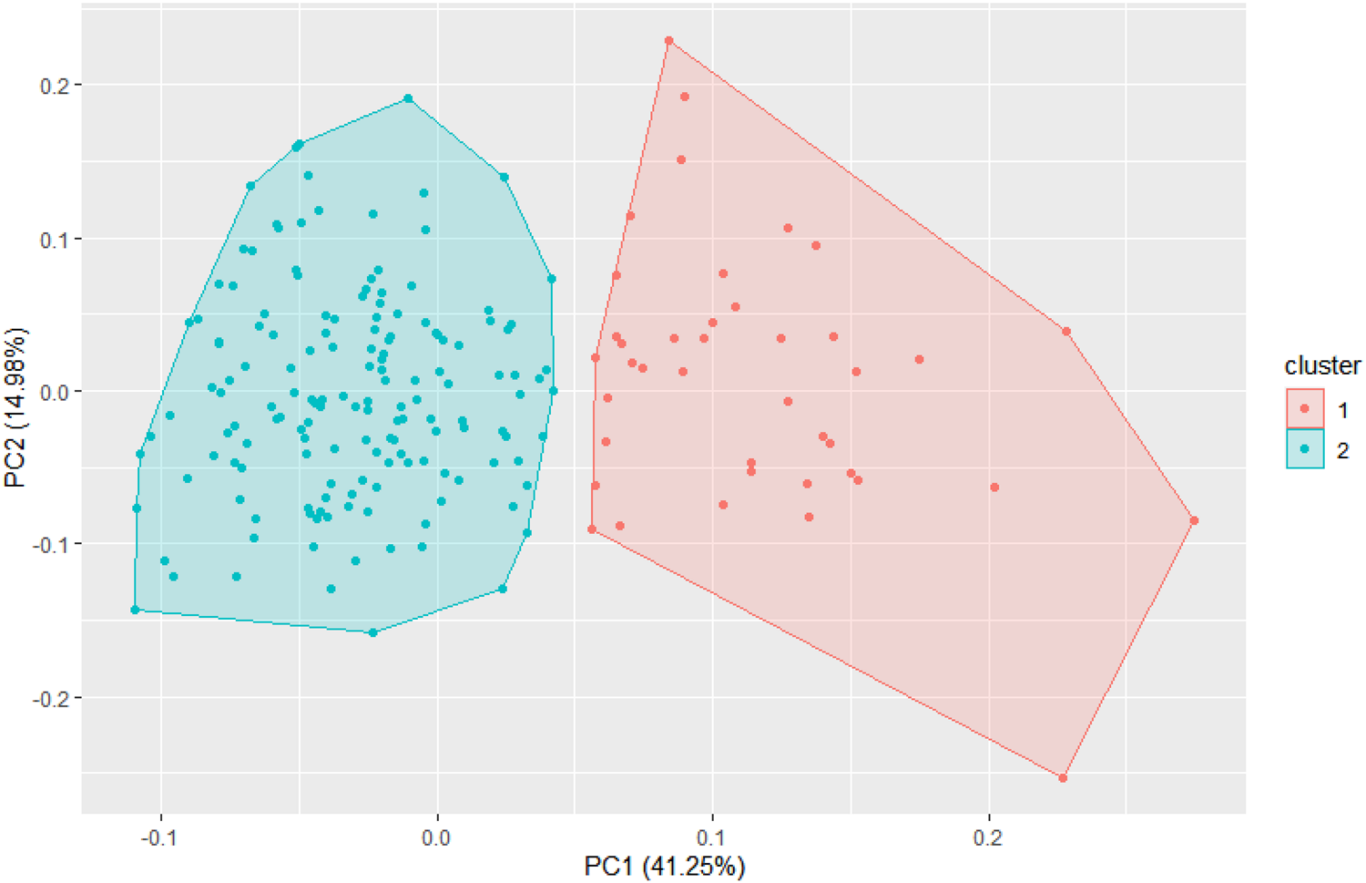
K-means cluster analysis for yield and biochemical traits evaluated in 10 populations of *Fragaria* × *ananassa* Dusch.

The optimal K value for the population was determined to be 2 according to the Elbow Method (Fig. 1). Two clusters were generated without overlapping in the K-means clustering (Fig. 2). The control ‘Camino Real’ and 40 seedling genotypes were in group 1. The control ‘Camarosa’ and 154 seedling genotypes were in group 2. Group 1 was better than group 2, according to the 5% confidence interval, for yield-related traits mass of commercial fruits (MCF), number of commercial fruits (NCF), and average mass commercial fruits (AMCF), as well as for the biochemical characteristics Ratio, ascorbic acid (AA), and anthocyanins (ANT). Contrarily, no significant difference in total pectin (TP) was measured between groups 1 and 2 (Table 1).

**Table 1:** Confidence interval of the mean values of the variables evaluated in the two K-means clusters with the respective number of genotypes (*p* < 0.05).

K-means clusters	1	2
Number of genotypes	41	155
MCF	[1474.90; 1831.82]	[324.81; 409.81]
NCF	[95.67; 117.69]	[24.55; 30.79]
AMCF	[14.24; 17.64]	[12.09; 12.95]
Ratio	[11.94; 14.00]	[9.09; 9.79]
TP	[1.73; 2.45]	[1.91; 2.19]
AA	[75.30; 81.86]	[67.80; 71.06]
ANT	[43.44; 50.20]	[35.71; 39.12]

*MCF* mass of commercial fruits (g plant^−1^), *AMCF* average mass of commercial fruits (g fruit^−1^), *NCF* number of commercial fruits (fruits plant^−1^), Ratio, soluble solids (Brix°)/titratable acidity (g citric acid 100 g^−1^ pulp); *TP* total pectin (g total pectin 100 g^−1^ pulp), *AA* ascorbic acid (mg ascorbic acid 100 g^−1^ pulp), and *ANT* anthocyanins (mg cyanidin-3-glucoside 100 g^−1^ pulp).

**Figure 3:**
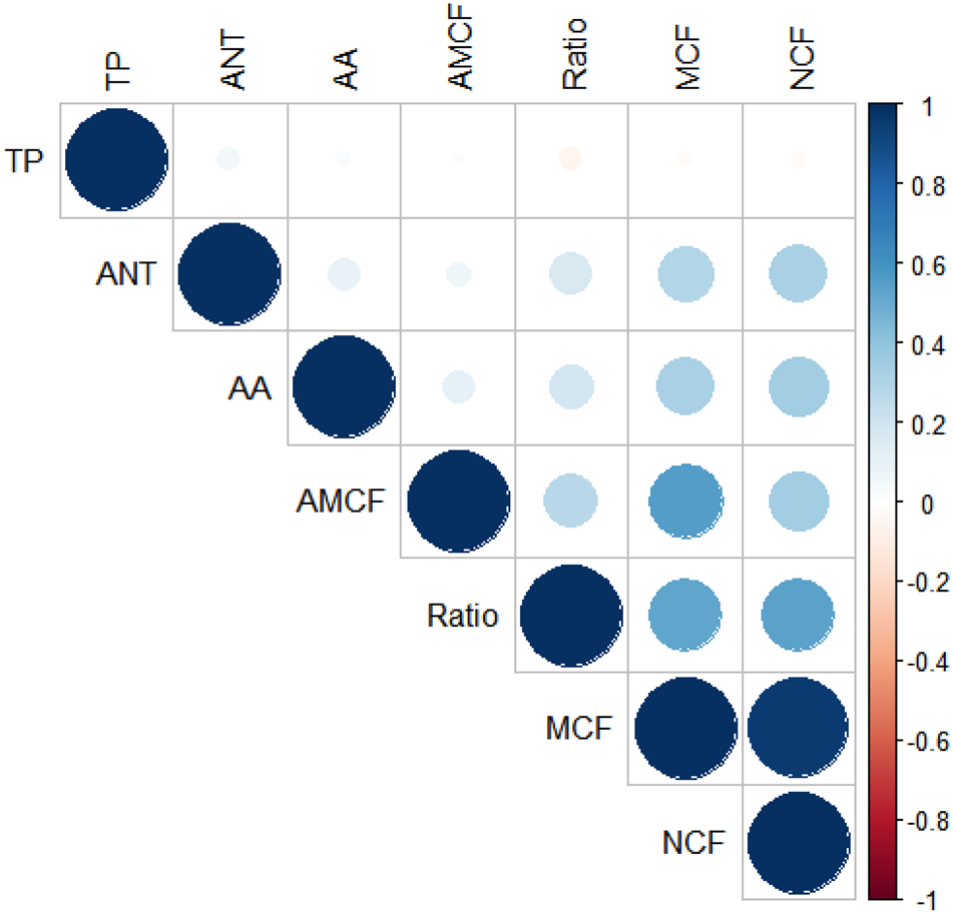
Pearson’s phenotypic correlations among yield and biochemical traits evaluated in 10 populations of *Fragaria* × *ananassa* Dusch. *NCF* number of commercial fruits (fruits plant^−1^), *AMCF* average mass of commercial fruits (g fruit^−1^), Ratio—soluble solids (Brix°)/titratable acidity (g citric acid 100 g^−1^ pulp), *TP* total pectin (g total pectin 100 g^−1^ pulp), *AA* ascorbic acid (mg ascorbic acid 100 g^−1^ pulp), and *ANT* anthocyanins (mg cyanidin-3-glucoside 100 g^−1^ pulp).

Twelve significant and positive correlations were found by the t-test (*p* < 0.05) among the 21 pairs of traits evaluated (Fig. 3). The most robust correlations were obtained for yield-related characteristics. High phenotypic correlations (r > 0.66) were measured between MCF and NCF (r = 0.96). Medium correlations (0.33 < r < 0.67) were measured between MCF and AMCF; MCF and Ratio; and NCF and Ratio, presenting r values of 0.55, 0.53, and 0.53, respectively. Low correlations (r < 0.34) were measured between NCF and AMCF (r = 0.34), between yield- and biochemical- related traits (MCF and AA (r = 0.32); MCF and ANT (r = 0.29); NCF and AA (r = 0.34); NCF and ANT (r = 0.32); and AMCF and Ratio (r = 0.27)), and between the biochemical traits Ratio and AA (r = 0.18) and Ratio and ANT (r = 0.32).

By unfolding the correlations through path analysis for a single causal diagram, direct and indirect effects for the independent trait MCF and the other characteristics were identified (Table 2). Biochemical-related traits had no direct or indirect effect on MCF. The NCF had a direct effect (0.88) on MCF and an indirect effect on AMCF (0.086). Contrarily, AMCF had an indirect effect on NCF (0.30) greater than the direct effect on itself (0.25). Ratio had a small direct effect on MCF, while presented a greater indirect effect on NCF (0.46) and AMCF (0.068).

**Table 2:** Direct (on the main diagonal) and indirect (on the upper and lower diagonals) effects of the independent variables on the mass of commercial fruits in 10 populations of *Fragaria* × *ananassa* Dusch.

	NCF	AMCF	Ratio	TP	AA	ANT
NCF	0.87785	0.08590	− 0.00086	− 0.00027	− 0.00011	− 0.00156
AMCF	0.29847	0.25266	− 0.00044	− 0.00009	− 0.00031	− 0.00028
Ratio	0.46526	0.06822	− 0.00016	− 0.00045	− 0.00059	− 0.00080
TP	− 0.02633	− 0.00253	0.00008	− 0.00091	− 0.00006	− 0.00023
AA	0.29847	0.02526	− 0.00031	0.00018	− 0.00314	− 0.00047
ANT	0.28969	0.01516	− 0.0028	0.00045	− 0.00031	− 0.00471
R^2^ = 0.98						
Residual effect = 0,14,752						

*NCF* number of commercial fruits (fruits plant^−1^), *AMCF* average mass of commercial fruits (g fruit^−1^), Ratio, soluble solids (Brix°)/titratable acidity (g citric acid 100 g^−1^ pulp); *TP* total pectin (g total pectin 100 g^−1^ pulp), *AA* ascorbic acid (mg ascorbic acid 100 g^−1^ pulp), and *ANT* anthocyanins (mg cyanidin-3-glucoside 100 g^−1^ pulp).

The total percentage gains obtained by the four indices under the four criteria (GV, h^2^, GCV, and EW) ranged from 366 to 386% (Table 3) for simultaneous selection of yield and biochemical traits. The Smith-Hazel index showed the highest total gains with 386%, for all criteria, followed by the Genotype-Ideotype distance index with 384% for GCV, the Mulamba and Mock index with 384% and 383% for EW and GCV, respectively, and by the Williams index with 380% for h^2^ (Table 3). Regarding yield and biochemical traits, the Mulamba and Mock index provided greater gains for the biochemical-related traits, in relation to h^2^ (86%) and GCV (81%), followed by the Genotype-Ideotype index, under the criteria h^2^ (79%) and GCV (75%). The Smith and Hazel index, despite having shown the greatest gains for yield traits, showed the lowest gains of biochemical traits, in which the four indices under the four criteria selected 53 genotypes (Table 4). From this total, 38 are located in group 1 and 15 in group 2, according to the K-means clustering. A total of 28 genotypes were selected by all indices for all criteria, in which only one belonged to group 1 of the K-means cluster analysis. Eleven genotypes were selected in some indices for all criteria and only in some criteria in other indices. Another eleven genotypes were selected in some indices for some criteria, and three genotypes were selected by some indices for all criteria.

**Table 3:** Estimates of percentage gains obtained by simultaneous selection with application of four indices based on four criteria of economic weights for seven traits evaluated in 10 populations of *Fragaria* × *ananassa* Dusch.

Traits	Smith and Hazel				Mulamba and Mock			
	GV %	h^2^%	GCV %	EW %	GV %	h^2^%	GCV %	EW %
MCF	162.5	162.5	162.5	162.5	163.5	142.5	151.9	162.7
AMCF	13.4	13.4	13.4	13.4	14.5	11.7	10.9	14.6
NCF	154.3	154.25	154.3	154.3	142.9	128.0	139.5	141.7
Total for yield traits	330.1	330.1	330.1	330.1	320.9	282.2	302.3	319.1
Ratio	26.9	26.9	26.9	26.9	25.9	28.6	28.8	27.8
TP	1.1	1.1	1.1	1.1	4.8	19.5	18.7	5.3
AA	11.5	11.5	11.5	11.5	10.9	12.0	10.2	11.5
ANT	16.8	16.8	16.8	16.8	17.2	26.3	23.4	20.0
Total for biochemical traits	56.4	56.4	56.4	56.4	58.9	86.3	81.1	64.5
Total	386.5	386.5	386.5	386.5	379.8	368.6	383.4	383.6

Traits	Williams				Genotype-ideotype			
	GV	h^2^	GCV	EW	GV	h^2^	GCV	EW
MCF	163.5	163.4	163.5	163.5	155.4	150.1	154.4	154.6
AMCF	14.5	13.8	14.3	14.3	13.9	11.2	9.7	14.0
NCF	142.9	144.9	143.8	143.8	137.4	137.1	145.0	136.2
Total of yield traits	320.9	322.0	321.5	321.5	306.6	298.4	309.0	304.8
Ratio	25.9	25.6	26.7	26.7	27.4	28.5	28.3	29.3
TP	4.8	2.9	3.0	3.0	5.6	13.7	11.6	6.0
AA	10.9	12.1	12.0	12.0	10.4	12.0	12.4	11.1
ANT	17.2	17.5	16.1	16.1	16.7	25.2	22.6	19.4
Total of biochemical traits	58.9	58.1	57.7	57.7	60.1	79.4	74.9	65.8
Total	379.8	380.1	379.3	379.3	366.7	377.8	383.9	370.5

*MCF* mass of commercial fruits (g plant^−1^), *AMCF* average mass of commercial fruits (g fruit^−1^), *NCF* number of commercial fruits (fruits plant^−1^), Ratio, soluble solids (Brix°)/titratable acidity (g citric acid 100 g^−1^ pulp); *TP* total pectin (g total pectin 100 g^−1^ pulp), *AA* ascorbic acid (mg ascorbic acid 100 g^−1^ pulp), and *ANT* anthocyanins (mg cyanidin-3-glucoside 100 g^−1^ pulp). *GV* genotypic variance, *h*^2^ herdability, *GCV* genetic coefficient variation and *EW* economic weight assigned by the breeder.

**Table 4:** Hybrids selected by Smith-Hazel, Mulamba and Mock, Williams, and Genotype-Ideotype indices and K-means clustering for yield and biochemical traits in 10 populations of *Fragaria* × *ananassa* Dusch.

Selection groups	Hybrids	K- means	MCF	NCF	AMCF	Ratio	TP	AA	ANT
Hybrids selected by all indices for all criteria	RVOT11	1	1097. 80	80.0	13.7	12.8	4.8	89.9	58.1
	RVOT12	1	1188.0	80.0	14.9	13.5	4.7	92.1	48.5
	RVOT21	1	1857.1	120.0	15.5	14.4	0.9	82.1	56.1
	RVOT22	1	2524.9	200.0	12.6	9.5	1.4	88.4	52.2
	RVFA02	1	1968.3	120.0	16.4	12.1	1.5	71.7	58.1
	RVFA04	1	1984.5	110.0	18.0	10.4	3.6	85.1	31.8
	RVFA14	1	1670.1	120.0	13.9	16.5	0.4	80.0	49.9
	RVFA16	1	1518.5	90.0	16.9	13.3	0.9	88.9	50.0
	RVSA14	1	1503.0	110.0	13.7	11.2	1.8	78.7	49.1
	RVDA01	1	1732.4	100.0	17.3	12.6	3.5	94.9	48.1
	RVDA12	1	1489.0	90.0	16.5	18.4	3.1	81.0	41.9
	RVCS01	1	1643.3	120.0	13.6	12.9	2.5	67.8	51.3
	RVCS04	1	2503.4	170.0	14.7	18.6	1.0	74.0	51.3
	RVCS07	1	2005.8	120.0	16.7	11.7	1.6	73.9	39.1
	RVCS09	1	1500.5	130.0	11.5	18.6	1.1	84.2	48.5
	RVCS10	1	2722.9	180.0	15.1	24.8	0.4	92.1	54.7
	RVCS11	1	1486.0	110.0	13.5	17.2	0.5	88.5	50.4
	RVTA12	1	2046.1	110.0	18.6	9.4	1.5	82.1	30.0
	RVTA16	1	2393.6	160.0	15.0	8.8	2.9	67.2	59.7
	RVTA20	1	1336.1	100.0	13.4	15.6	3.1	85.7	44.3
	RVCA02	1	1042.1	70.0	14.9	15.1	1.7	65.7	61.8
	RVCA05	2	1162.9	60.0	19.4	7.8	1.3	61.3	47.9
	RVCA06	1	2247.4	150.0	16.3	12.5	1.9	56.0	63.0
	RVCA08	1	1133.8	80.0	14.2	15.3	1.5	68.9	27.4
	RVCA10	1	1042.9	70.0	14.9	13.2	2.7	80.0	37.5
	RVCA11	1	924.4	70.0	13.2	17.4	2.9	87.1	29.9
	RVCA14	1	1037.9	60.0	17.3	11.8	3.2	78.4	61.1
	RVCA16	1	2109.9	120.0	17.6	13.1	2.6	80.2	49.9
Hybrids selected by some indices for all criteria and by other indices for only a couple of criteria	RVFS01	1	2060.9	130.0	15.9	15.1	1.6	92.5	32.5
	RVFS24	1	1302.5	100.0	13.0	14.6	3.0	89.8	28.4
	RVDS24	1	1350.0	110.0	12.3	7.9	1.6	80.0	54.2
	RVSA08	1	1388.0	110.0	12.6	14.3	3.5	68.4	25.5
	RVDA03	1	1532.6	100.0	15.3	11.8	2.6	91.3	38.5
	RVDA04	1	3370.7	200.0	16.9	11.1	2.6	96.6	41.3
	RVDA11	1	2158.7	150.0	14.4	14.6	0.4	59.9	65.1
	RVDA18	1	2987.3	60.0	49.8	13.2	1.9	61.2	31.1
	RVCS03	2	753.7	60.0	12.6	12.5	1.7	75.0	44.6
	RVCS12	2	723.5	50.0	14.5	15.7	1.1	65.5	25.5
	RVTS08	1	1094.6	80.0	13.7	12.6	3.0	82.4	45.2
Hybrids selected by some indices for all criteria	RVDS14	1	1372.4	90.0	15.2	11.1	1.5	62.5	47.6
	RVCS14	2	668.1	60.0	11.1	14.8	1.7	70.0	38.6
	RVCA01	2	900.2	60.0	15.0	10.7	1.8	74.1	49.2
Hybrids selected by some indices for some parameters	RVOT28	2	894.6	70.0	12.8	10.5	4.3	73.7	41.3
	RVDS26	1	1126.1	80.0	14.1	13.8	3.6	82.5	61.3
	RVFA01	2	741.1	40.0	18.5	6.4	2.9	75.3	39.5
	RVSA12	2	929.1	70.0	13.3	10.9	2.9	55.9	48.1
	RVTS01	2	1112.3	80.0	13.9	8.5	3.0	75.6	32.0
	RVTS15	2	1070.9	90.0	11.9	7.8	0.9	77.9	56.3
	RVCA03	2	744.5	60.0	12.4	12.1	1.7	71.7	50.4
	RVCA07	2	451.5	40.0	11.3	10.3	2.9	58.2	51.4
	RVCA12	2	720.3	50.0	14.4	14.8	2.1	65.8	51.8
	RVCA13	2	170.9	10.0	17.1	11.3	3.1	70.6	50.0
	RVCA15	2	597.6	30.0	19.9	12.5	3.5	55.9	31.6
Controls	Camarosa	2	776.2	45.0	16.9	10.8	1.9	74.5	39.8
	Camino Real	1	1173.0	64.0	18.3	11.4	2.2	77.4	42.3

*MCF* mass of commercial fruits (g plant^−1^), *AMCF* average mass of commercial fruits (g fruit^−1^), *NCF* number of commercial fruits (fruits plant^−1^), Ratio, soluble solids (Brix°)/titratable acidity (g citric acid 100 g^−1^ pulp); *TP* total pectin (g total pectin 100 g^−1^ pulp), *AA* ascorbic acid (mg ascorbic acid 100 g^−1^ pulp), and *ANT* anthocyanins (mg cyanidin-3-glucoside 100 g^−1^ pulp).

The crosses ‘Camarosa’ × Aromas’ and ‘Camarosa’ × ‘Sweet Charlie’ stood out, with 14 and nine selected hybrids, respectively. The other crosses showed the following number of selected hybrids: ‘Dover’ × ‘Aromas’—6, ‘Oso Grande’ × ‘Tudla’—5, ‘Festival’ × ‘Aromas’—5, ‘Aromas’ × ‘Sweet Charlie’—3, ‘Tudla’ × ‘Aromas’—3, ‘Dover’ × ‘Sweet Charlie’—3, ‘Tudla’ × ‘Sweet Charlie’ – 3, and ‘Festival’ × ‘Sweet Charlie’—2.

**Figure 4:**
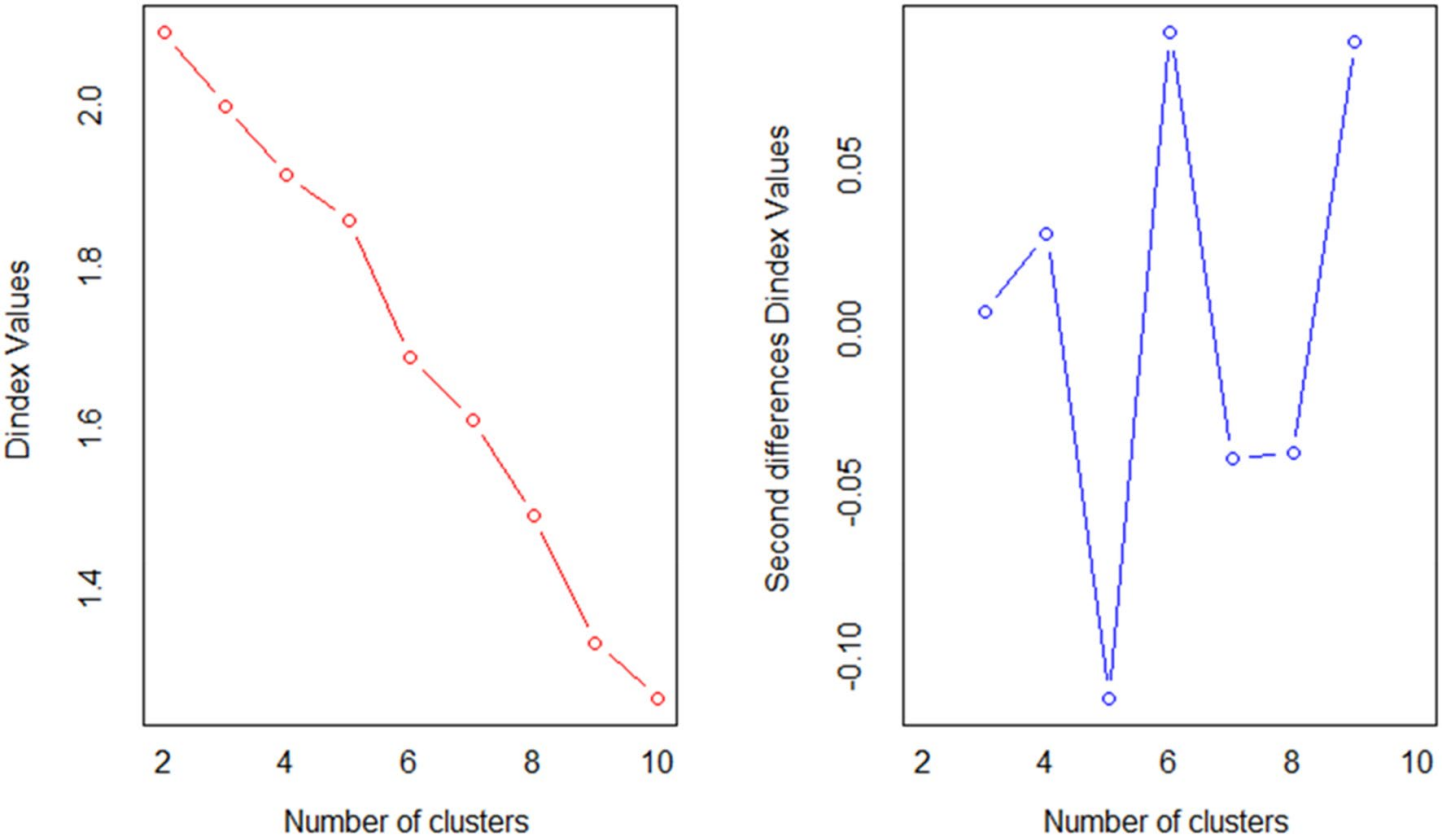
Dindex graphic for determining the best number of clusters in 53 selected genotypes and two controls of *Fragaria* × *ananassa* Dusch.

**Figure 5:**
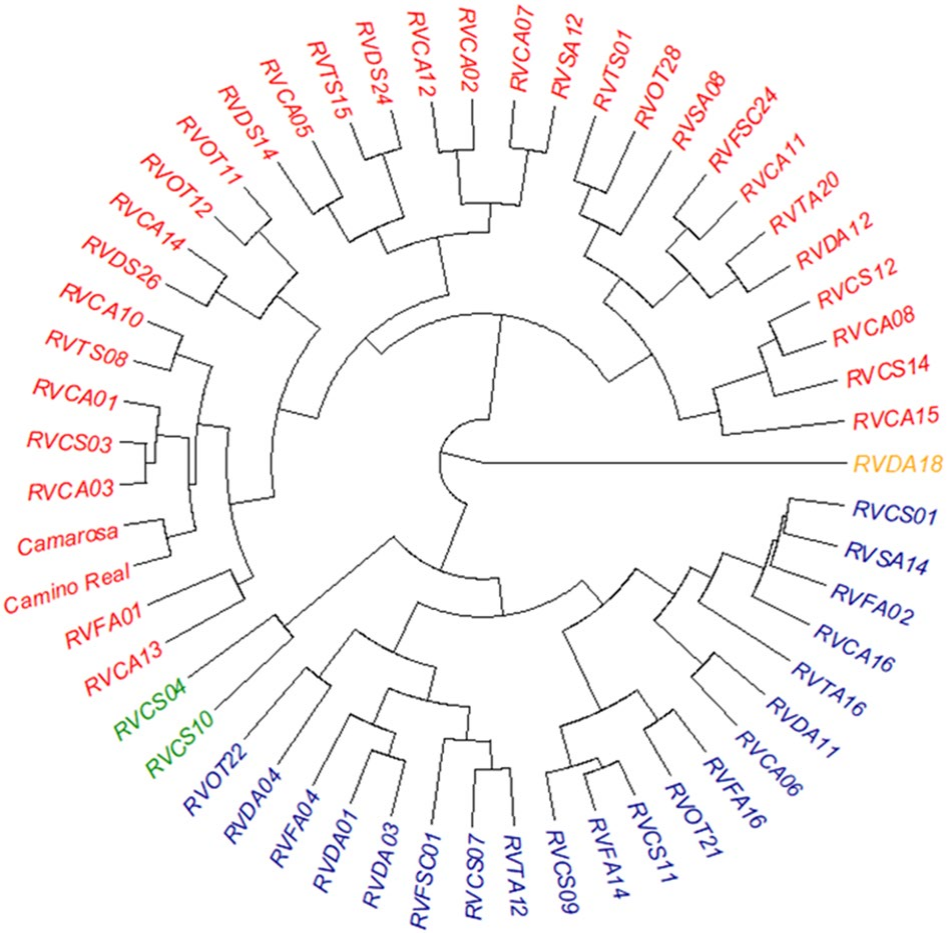
Circular dendrogram based on yield and biochemical traits of the 53 selected genotypes and two controls of *Fragaria* × *ananassa* Dusch.

According to Dindex (Fig. 4), genotypes can be grouped into four groups by a significant knee in the plot of index values against the number of clusters. The circular hierarchical dendrogram (Fig. 5) obtained from the analysis of the 53 genotypes selected by the indices and the controls (‘Camarosa’ and ‘Camino Real’), generated groups with 32, 20, 2 and 1 genotypes, whose cophenetic correlation value was 0.827 (*p* < 0.05).

## Discussion

Brazilian strawberry production depends almost entirely on cultivars developed in foreign breeding programs that, due to aspects related to genotype × environment interactions, may present lower yield, lower biochemical quality, greater susceptibility to pests and diseases, increasing production costs^5^. Nonetheless, these imported cultivars have the potential to be explored in intraspecific crosses aiming to express the existing variability in the species^13,27^.

Strawberry is an octoploid species that has gone through various levels of ploidization throughout evolutionary history^28^. Strawberry also harbors millions of DNA variants of the subgenomes of the species that gave rise to actual strawberry fruit^29^. In general, strawberry presents great variability in hybrids obtained from crosses, which favors the selection of new cultivars^30^. Significant variability with identification of superior hybrids has been found in phenotypic analyses for yield and physicochemical traits in populations obtained from crosses between commercial strawberry cultivars in Brazil^9,12,13,31–33^. In addition, genetic studies with hybrids and commercial cultivars based on molecular markers have shown that the germplasm of the Brazilian strawberry breeding program has genetic variability and divergence; therefore, it has a high potential for launching new cultivars^9,34^.

For the population analyzed in this study, the Elbow method established two clusters, which presented no overlap in the K-means clustering, showing variability in the analyzed population and complete dissimilarity between the two groups formed. The highest phenotypic correlation for the independent variable mass of commercial fruits (MCF) was obtained for the number of commercial fruits (NCF) (0.96), which had a high direct effect (0.88) in the path analysis. The average mass of commercial fruits (AMCF), which also had a medium and positive correlation (0.55) with the MCF, demonstrated in the path analysis that its indirect effect (0.29) on NCF is superior to the direct effect (0.25). Diel et al.^35^ found a direct effect of the total number of fruits (0.81), and an indirect effect of the mass of commercial fruits, via the total number of fruits (0.71), while the average fruit mass showed a direct relationship of 0.22. Authors results corroborate with our study and these positive findings suggest that direct selection via number of commercial fruits has a greater effect on yield and indirectly benefits the average mass of commercial fruits.

The balance between soluble solids and titratable acidity (Ratio) represents the equilibrium between sweetness and acidity. This balance combined with aroma and other biochemical traits makes up flavor, which has great importance in sensory perception and consumer preference^5,6^. In the present study, Ratio showed a moderate and positive phenotypic correlation with the mass of commercial fruits (0.52) and number of commercial fruits (0.53); however, when unfolding this correlation, a negative direct effect was observed, while the indirect effect was positive via NCF. In agreement with the present study, Diel^35^ found a negative direct effect (− 0.10) and a positive indirect effect (0.15) of Ratio via the total number of fruits on the total fruit mass. Direct effects of the number of strawberry fruit on production per plant were also reported by Ara et al.^36^ and Garg^11^, while Sighn et al.^37^ stated that the greatest direct positive effects came from flower number and fruit length. These results evince that the selection of strawberry genotypes for mass of commercial fruits can be directly performed via the number of commercial fruits and that genotypes with numerous fruits, but of medium size, tend to have a better Ratio than genotypes with large fruits.

Selecting genotypes that balance yield and biochemical traits simultaneously is a complex task^10^. The use of selection indices, both parametric and non-parametric, has been useful to identify more balanced hybrids of diverse crops, such as sweet potato^26^, alfalfa^38^, soybean^25,39,40^, potato^41^, maize^42^, acai^43^, passion fruit^23,44^, and, more recently, strawberry^13,27^.

In the present study, the Mulamba and Mock and Genotype-Ideotype indices were more sensitive to the use of different criteria, showing greater differences between gains. Cruz et al.^45^ recommend the use of statistics obtained from the analysis of experimental data as economic weights (EW) since it relates to the genotypic variance, they are dimensionless and maintain a certain proportionality among the evaluated traits. In the present study, the greatest gains for yield traits were obtained by the Smith and Hazel index (330.14%); however, it showed no difference between the statistical criteria or assigned weights. Contrarily, the greatest gains for the biochemical-related traits were obtained by the Mulamba and Mock and Genotype-Ideotype indices, under the criteria of h^2^ and GCV with 86.34% and 81.06%; 79.41% and 74.87%, respectively. Vieira et al.^27^, evaluating strawberry genotypes, also reported the greatest increments for yield traits with the Smith and Hazel index and for biochemical characteristics applying the Mulamba and Mock index. It occurs because parametric tests use the distribution parameters to calculate the statistics, while non-parametric tests use ranks assigned to ordered data and are uninfluenced by the probability distribution of the data evaluated^46^. Thus, the non-parametric Mulamba and Mock index is less sensitive, mathematically, to traits that present wide variance, such as number of fruits.

From the 194 genotypes analyzed, 28 were selected for all indices, under all criteria, in which 27 belong to group 1 of the K-means clustering. The use of different indexes and criteria tend to present very similar results for the initial positions of the selected genotypes. Bernardo et al.^47^ analyzed studies in several agronomic crops and concluded that, if the population is large enough, any selection index applied judiciously is useful for the simultaneous improvement of multiple traits, regardless of the method used. Nevertheless, the indices start to select different hybrids for the different criteria with the progress of positions.

The crosses with the highest number of selected hybrids were ‘Camarosa’ × ‘Aromas’ and ‘Camarosa’ × ‘Sweet Charlie’. Similarly, Galvão et al.^28^ identified the best hybrids for yield traits in the cross between ‘Camarosa’ × ‘Aromas’. Camarosa has been reported as a highly productive cultivar, with large, firm, and tasty fruits^48^, being one of the most planted short-day cultivars in the world^49^. The presence of large number of favorable alleles in ‘Camarosa’ and ‘Aromas’^33^ and their high productive potential^50,51^ make them promising parents for strawberry breeding programs^5^. Camargo et al.^32^ also found and selected the best hybrids coming from the crosses ‘Camarosa’ × ‘Aromas’ and ‘Camarosa’ × ‘Sweet Charlie’, concerning biochemical traits.

The dendrogram generated from the 53 selected genotypes led to the formation of five groups, demonstrating that this population still has variability that can be further investigated.

## Conclusion

K-means clustering, correlation analysis, and path analysis complement the use of selection indices, leading to the selection of hybrids with better balance between yield- and biochemical-related traits in strawberry. This combined approach is more promising than the direct selection based on only one or a few traits. Furthermore, the multivariate analysis methods were efficient in selecting strawberry genotypes for multi-characters.

The number of commercial fruits was more relevant to the mass of commercial fruits than the average mass of commercial fruits. Therefore, NCF is a trait of greater importance for the selection of strawberry genotypes aiming at yield. The Smith and Hazel index showed the greatest gain for yield traits. Possibly because it is mathematically more influenced by characteristics with greater variability such as yield. The Mulamba and Mock and Genotype-Ideotype indices, both non-parametric, showed the highest estimated gains for biochemical traits under the criteria of h^2^ and GCV. The crosses with the highest number of selected hybrids were ‘Camarosa’ × ‘Aromas’ and ‘Camarosa’ × ‘Sweet Charlie’. The selected population of 53 hybrids still has variability with potential to be exploited.

## Material and methods

The material and methods of our study was performed in accordance with the relevant guidelines and regulations. Plant material and replications followed the regulations of the Ministry of Agriculture, Cattle and Supplying of Brazil.

### Plant material

Ten populations were obtained from biparental crosses among strawberry cultivars traditionally grown in South America (Table 5). All parents are public commercial cultivars available at the Brazilian Agricultural Research Corporation (EMBRAPA) from the Ministry of Agriculture, Cattle and Supplying of Brazil, and they were grown with Multiplanta Tecnologia Vegetal (Andradas, MG, Brazil). Parents are short-day cultivars based on photoperiod responses, except by Aromas which is a day-neutral cultivar^52^. Hybridization was performed following Chandler et al.^53^. The choice between cultivars to carry out the crosses to obtain segregating populations was based on the genetic dissimilarity study carried out by Morales et al.^34^. After crossing, achenes present in the fruits were removed and germinated in vitro, as described by Galvão et al.^31^. At 60 days after germination, the seedlings were transplanted to 72-cell polypropylene trays containing biostabilized substrate. A total of 2000 plants (about 200 seedlings per population) were transplanted to low-tunnel-covered beds in an augmented block design. Seedlings were transplanted in April 2015 and the genotypes evaluated until November of the same year. Based on agronomic (total and commercial fruit production, average fruit mass), phytosanitary [no symptoms of anthracnose (*Colletotrichum acutatum* and *C. fragariae*), *Botrytis cinerea* and *Mycosphaerella fragariae*), SS (Content of soluble solids above 8° Brix)], firmness traits and distribution of production on cycle, 194 genotypes were selected, grown in the greenhouse, cloned, and transplanted to the experimental field. Strawberry runners were transplanted in trays with substrates to obtain seedlings in sufficient numbers to be used as replications.

**Table 5:** Intraspecific crosses used to obtain 10 segregating strawberry (*Fragaria × ananassa* Dusch.) populations.

Population	Parents	
	**♀**	**♂**
1—DA	Dover	Aromas
2—CA	Camarosa	Aromas
3—DS	Dover	Sweet Charlie
4—OT	Oso Grande	Tudla
5—FS	Florida Festival	Sweet Charlie
6—AS	Sweet Charlie	Aromas
7—TA	Tudla	Aromas
8—TS	Tudla	Sweet Charlie
9—CS	Camarosa	Sweet Charlie
10—FA	Florida Festival	Aromas

### Experimental area

The experimental area is located in the city of Guarapuava, Paraná, Brazil (25° 23′ 36″ S and 51° 27′ 19″ W). The area has a humid mesothermal subtropical climate, type Cfb, with moderate winter and summer with average temperatures around 22 °C according to the Köppen's classification^54^. The soil is classified as a typical dystroferric Bruno Latosol^55^.

Seedlings were obtained from the stolons emitted by the parent plants, kept in a greenhouse. Rooting took place in 46-cell polypropylene trays filled with commercial substrate. At 50 days after planting, seedlings were transplanted in the experimental area, with evaluations occurring between May and November 2016.

Strawberry transplanting was performed in a low-tunnel system 0.8 m high with beds 1 m wide × and 0.25 m high surface covered with a black polyethylene film 30-µm thick. To cover the tunnels, 120-µm thick transparent polyethylene film was used. The plant spacing was 0.30 × 0.30 m between plants and 0.40 m between rows.

Beds were fertilized with 1,650 kg ha^−1^ of simple superphosphate, 250 kg ha^−1^ of potassium chloride, and 295 kg ha^−1^ of urea, based on the soil chemical analysis in accordance with the recommendations for the strawberry crop^52^. Nutritional replacement was performed via fertigation twice a week. Irrigation water was provided using a micro-drip system and followed the crop water demand. Additionally, for phytosanitary preventive control, applications of Thiamethoxam and Azoxystrobin + Difenoconazole were carried out. Strawberry fruits were harvested at maturity stage when 75% of fruit were red.

The experiment was conducted using the randomized block design with three replications and ten plants per plot. There was a total of 194 F_1_ experimental hybrids and two commercial controls ('Camarosa' and 'Camino Real').

### Yield and biochemical traits evaluated

Traits that showed significant differences in the analysis of variance were used in the further analyses, namely: mass of commercial fruits (MCF) (g plant^−1^), number of commercial fruits (NCF) (fruit plant^−1^), average mass of commercial fruits (AMCF) (g fruit^−1^), ratio between soluble solids (SS) (Brix°) and titratable acidity (TA) (g citric acid 100 g^−1^ pulp (Ratio), total pectin (g total pectin 100 g^−1^ pulp), ascorbic acid content (AA) (mg ascorbic acid 100 g^−1^ pulp), and anthocyanin content (ANT) (mg cyanidin-3-glucoside 100 g^−1^ pulp).The biochemical traits were assessed in samples of commercial ripe strawberries (above 10 g), stored at − 2 °C right after harvest. Strawberries were thawed, crushed, and homogenized. Using the homogenized pulp, soluble solids content was measured with an Optech bench refractometer. Titratable acidity was determined by the titration method, with aliquots of 10 g of strawberry pulp plus 100 mL of distilled water 0.1 mol L^−1^NaOH standard solution up to pH 8.2, which corresponds to the turning point of phenolphthalein^56^. The total pectin was determined by the method described by McCready and McComb^57^, and calorimetrically determined while using the carbazole reaction, according to the methodology that was described by Bitter and Muir^58^. Ascorbic acid was obtained by the standard titration method of the Association of Official Analytical Chemists (AOAC), modified by Benassi and Antunes^59^. Whereas anthocyanin was determined by the differential pH method described by Giusti and Wrosltad^60^, with adaptations for strawberry. All biochemical analyses were performed in triplicates.

### Statistical analyses

The variability of the 194 genotypes/hybrids and the two controls was analyzed using the R software (http://cran-rc3sl.ufpr.br). First, the number of clusters was determined by the Elbow Method using the factextra v.1.0.7 package^61^. A graph was used to indicate the ideal cluster number to represent a data set, where the value of “K” to be used is the point of the curve that looks like an elbow (inflection). Subsequently, the K-means non-hierarchical cluster analysis was performed based on the Euclidean distance, with the stats R Core Team^62^, dplyr v.0.8.5^63^, ggplot2^64^, and ggfortify^65^ packages. The relationship between traits was performed using a Pearson correlation map thoughtout the corrplot v.0.84 package^66^, while a path analysis was performed with agricolae v.1.3-2^67^. The fenotypic correlations were classified as high (r > 0.66), medium (0.33 < r < 0.67) and low (r < 0.33)^68^.

Variance component analysis was performed with the Genes software^69,70^ to estimate genotypic variance (GV), heritability (h^2^), and genotypic coefficient variation (GCV). Economic weights (EW) were assigned (Table 6). Subsequently, two parametric indices, the classic index from Smith^17^ and Hazel^18^ and the base index^19^, and two non-parametric indices, the rank-sum-based index^20^, and the genotype-ideotype distance index^21^ were used to select.

**Table 6:** Economic weights criteria used in the application of selection indices for trait analysis in 10 populations of *Fragaria* × *ananassa* Dutch.

Variables	Parameters			
	Genotypic variance	Herdability	GCV	Assigned weight
MCF	462,808.65	87.59	104.89	500
AMCF	10.19	53.84	24.24	100
NCF	1799.28	93.46	93.61	100
Ratio	9.72	82.91	30.49	100
TP	1.53	87.87	59.97	50
AA	128.37	82.92	15.86	25
ANT	191.17	93.73	34.99	25

*MCF* mass of commercial fruits (g plant^−1^), *AMCF* average mass of commercial fruits (g fruit^−1^), *NCF* number of commercial fruits (fruits plant^−1^), Ratio, soluble solids (Brix°)/titratable acidity (g citric acid 100 g^−1^ pulp); *TP* total pectin (g total pectin 100 g^−1^ pulp), *AA* ascorbic acid (mg ascorbic acid 100 g^−1^ pulp), and *ANT* anthocyanins (mg cyanidin-3-glucoside 100 g^−1^ pulp).

The genotypic aggregate (H) in the classic Smith-Haze index it is obtained by the expression ${\text{H}}\, = \,{\text{a}}_{{1}} {\text{g}}_{{1}} \, + \,{\text{a}}_{{2}} {\text{g}}_{{2}} \, + \cdots {\text{a}}_{{\text{n}}} {\text{g}}_{{\text{n}}}$, where “a” is the n × 1 dimension vector of the economic weights and “g” is the p × n dimension matrix of unknown genetic values of the “n” traits for the “p” families or progenies evaluated. The index (I) consists of a linear combination of the “x” values measured of each trait, pondered by a coefficient. It is obtained by the expression: ${\text{I}}\, = \,{\text{b}}_{{1}} {\text{x}}_{{1}} \, + \,{\text{b}}_{{2}} {\text{x}}_{{2}} \, + \cdots {\text{b}}_{{\text{n}}} {\text{x}}_{{\text{n}}} .$, where the coefficient “b” is an (n × 1) vector estimated from the expression b = P^−1^ Ga, where “P^−1^” is the inverse of the phenotypic covariance matrix; “G” is the genetic covariance matrix and “a” is the (n × 1) vector of the economic weights assigned to the traits^17,18^.

This index of Mulamba and Mock is obtained by the expression: ${\text{I}}\, = \,{\text{r}}_{{1}} \, + \,{\text{r}}_{{2}} \, + \cdots + \,{\text{r}}_{{\text{n}}}$ , where “I” is the index value for a given individual, r_j_ is the rank of an individual in relation to the j-th variable, and “n” is the number of traits considered in the index. This procedure allows the ranking order of traits to have different weights, as specified by the breeder. Thus, we have that ${\text{I}}\, = \,{\text{p}}_{{1}} {\text{r}}_{{1}} \, + \,{\text{p}}_{{2}} {\text{r}}_{{2}} \, + \cdots + \,{\text{p}}_{{\text{n}}} {\text{r}}_{{\text{n}}}$, with p_j_ being the economic weight attributed by the breeder to the j-th trait^20^.

To obtain the genotype-ideotype index, the values that will express the distance between genotypes and the ideotype are calculated by the expression: I_DGI_ = √1/n Σ(y_ij_ − vo_j_)^2^. The best genotypes were identified, and selection gains were estimated based on I_DGI_. Based on the values of the ideotype (Y_ij_), the principal components analysis was performed to obtain the eigenvalues and eigenvectors associated with the correlation matrix between the analyzed variables. The distances of the genotypes in relation to the ideotype were estimated. This process allows the selection of genotypes closer to the optimal pattern defined by the breeder (ideotype)^21^.

Selection gains [SG (%)] in the base index^19^ were estimated with the following expression: SG (%) = 100 h^2^ (Xs − Xo)/Xo, where Xs is the average genotypic value of selected hybrids, Xo is the average genotypic value of all hybrids, and h^2^ is the heritability of the trait of interest. Heritability was obtained by the ratio between genotypic and phenotypic variance, as $h^{2} = \hat{\sigma }_{g}^{2} /\hat{\sigma }_{p}^{2}$, where $\hat{\sigma }_{g}^{2}$ is the genotypic variance and $\hat{\sigma }_{p}^{2}$ is the phenotypic variance^19^.

Lastly, the optimal number of clusters was identified by Dindex index with R package NbClust^71^ to generate a circular hierarchical dendrogram created with all selected hybrids and controls, in all parameters and indices using the R packages vegan v.2.5–6^72^**,** for the standardization of data, ape v.5.0^73^, and cluster v.2.1.0^74^.

## Data Availability

The datasets used and/or analyzed during the current study is available from the corresponding author on reasonable request.
